# Graph-based EEG approach for depression prediction: integrating time-frequency complexity and spatial topology

**DOI:** 10.3389/fnins.2024.1367212

**Published:** 2024-04-03

**Authors:** Wei Liu, Kebin Jia, Zhuozheng Wang

**Affiliations:** ^1^Faculty of Information Technology, Beijing University of Technology, Beijing, China; ^2^Beijing Laboratory of Advanced Information Networks, Beijing, China; ^3^Beijing Key Laboratory of Computational Intelligence and Intelligent System, Beijing University of Technology, Beijing, China

**Keywords:** EEG signal, depression prediction, graph convolutional network, time-frequency complexity, spatial topology, brain network

## Abstract

Depression has become the prevailing global mental health concern. The accuracy of traditional depression diagnosis methods faces challenges due to diverse factors, making primary identification a complex task. Thus, the imperative lies in developing a method that fulfills objectivity and effectiveness criteria for depression identification. Current research underscores notable disparities in brain activity between individuals with depression and those without. The Electroencephalogram (EEG), as a biologically reflective and easily accessible signal, is widely used to diagnose depression. This article introduces an innovative depression prediction strategy that merges time-frequency complexity and electrode spatial topology to aid in depression diagnosis. Initially, time-frequency complexity and temporal features of the EEG signal are extracted to generate node features for a graph convolutional network. Subsequently, leveraging channel correlation, the brain network adjacency matrix is employed and calculated. The final depression classification is achieved by training and validating a graph convolutional network with graph node features and a brain network adjacency matrix based on channel correlation. The proposed strategy has been validated using two publicly available EEG datasets, MODMA and PRED+CT, achieving notable accuracy rates of 98.30 and 96.51%, respectively. These outcomes affirm the reliability and utility of our proposed strategy in predicting depression using EEG signals. Additionally, the findings substantiate the effectiveness of EEG time-frequency complexity characteristics as valuable biomarkers for depression prediction.

## 1 Introduction

Life and work pressures are increasing, leading to increased mental stress and depression (Cunningham et al., 2018; Mokdad et al., 2018). Depression, a stress-related psychological condition, profoundly affects individuals’ daily lives. Globally, depression affects over 350 million people, with the World Health Organization (WHO) predicting it to become the most prevalent mental disorder by 2030 (Mathers and Loncar, 2006; World Federation for Mental Health, 2012; World Health World Health Organization, 2017; Lanillos et al., 2020). In China, approximately 95 million individuals are affected by depression, with an estimated 16% of the population expected to experience depression in their lifetimes. Severe depression can lead to suicide, with studies indicating a strong association between depression and suicidal behavior (Hawton et al., 2013). However, the current diagnostic model for depression lacks clinical effectiveness, making the diagnostic process challenging and subjective (Maj et al., 2020). Limited awareness, untrained healthcare professionals, and inaccurate diagnoses contribute to the fact that half of individuals with depression do not receive treatment. Prompt and accurate diagnosis is crucial for effective depression management, emphasizing the urgent need to further understand its etiology and pathogenesis.

EEG has proven to be a valuable diagnostic tool for various conditions due to its non-invasive and cost-effective nature. This bioelectrical signal, generated by the human brain, records ongoing and irregular potential changes during neural activity. It encapsulates rich physiological and psychological data, making it a promising biomarker and diagnostic aid for neurological disorders such as depression, epilepsy, seizures, Alzheimer’s, and Parkinson’s, as well as for emotional analysis (Subasi and Ercelebi, 2005; Jurysta et al., 2010; Kayser and Tenke, 2010; Acharya et al., 2013; Liao et al., 2017; Bhat et al., 2018; Sharma et al., 2018; Acharya et al., 2019; Gao et al., 2021; Gu et al., 2021; Saeidi et al., 2021). Exploring deeper into and extracting key features from EEG signals of depressed patients can facilitate their identification. Consequently, research efforts have increasingly focused on effectively extracting characteristic values from these signals in recent years.

With the advancement of technology, EEG data has been widely collected, and numerous studies have been conducted on the classification and recognition of EEG features associated with depression, utilizing various classification techniques and specific feature selection methods. For instance, Hosseinifard et al. (2011) utilized power spectrum features across different frequency bands for depression recognition. They employed a genetic algorithm to refine the most pertinent features and achieved an impressive accuracy of 88.6% when combined with a support vector machine (SVM) classifier. This approach provides a promising framework for depression detection based on spectral analysis. Ay et al. (2019) introduced a deep hybrid model, incorporating both convolutional neural networks (CNN) and long short-term memory (LSTM) structures for depression detection through EEG signals. This model demonstrated classification accuracies exceeding 85% for both the left and right hemispheres of the brain. Mohammadi et al. (2015) utilized data mining techniques, incorporating linear discriminant analysis (LDA), a genetic algorithm for feature mapping and selection, and a decision tree-based classification approach. Their method, utilizing EEG signals, achieved an accuracy of 80%. Separately, Li et al. (2022) introduced an automatic depression detection framework built upon a two-stage feature selection method. This framework employed EEG signals, incorporating the Pearson correlation coefficient and recursive feature elimination techniques, achieving a remarkable accuracy of 98.95% when using SVM with derived features from the alpha and beta frequency bands. Additionally, Liao et al. (2017) developed a method for depression detection from multi-channel EEG signals. They employed a spectral-spatial feature extractor known as the kernel eigen-filter bank, achieving a classification accuracy of 80% using the SVM approach. Wang Y. et al. (2021) employed the intrinsic time scale decomposition method to decompose each EEG record into several components, thereby obtaining feature vectors. They modified the original loss function softmax to L-Softmax in the time convolution network, achieving an accuracy of 86.87%. Shen (2021) analyzed EEG data from subjects with depression and optimized the lead space of the EEG signal through the use of loss minimization and adaptive lead weighting methods. By utilizing the spatial characteristics of EEG signals, they predicted depression, achieving an accuracy rate of 68.13%.

Most previous studies only considered temporal or spatial characteristics, and the accuracy of depression prediction algorithms is still not ideal. The EEG signals of depression are not only significantly different from those of normal subjects in temporal characteristics but also have a strong synchronous coupling relationship in space. Therefore, unique information on depression EEG can be extracted from the perspective of space–time relationship to enhance the prediction accuracy.

Currently, there has been a significant shift in research focus towards exploring the connectivity and structural characteristics among brain regions. This shift has been marked by an emphasis on generating functional connectivity matrices, which serve as a crucial link to subsequent discussions about depression recognition. In 2020, Rong (2020) developed a convolutional neural network (CNN) recognition model specifically for mild depression, utilizing functional connectivity matrices. This model achieved a recognition rate of 80.74%. Additionally, Chen (2020) conducted a study in 2020 examining brain functional networks through various functional connectivity approaches. Their findings revealed that coherent brain functional networks, combined with support vector machines (SVMs), yielded the best dichotomous recognition results, achieving an accuracy of 90%. Wang D. et al. (2021) employed a semi-supervised learning approach, combining self-organizing incremental neural networks with graph convolutional networks (GCNs) for self-training. This method aimed to expand the training set and achieved a classification accuracy of 92.23% on the public MODMA dataset in a cross-subject scenario, requiring only 600 labeled samples. Separately, Chen et al. (2022) utilized graph pooling operations alongside a self-attention mechanism. In their construction of the adjacency matrix, they integrated prior knowledge, incorporating global connections. This approach resulted in an accuracy of 84.91% in the cross-subject task on the MODMA dataset. More recently, Zhu et al. (2022) introduced the concept of a learning weight matrix into the input layer of a graph convolutional neural network (GCN). This innovation aimed to optimize the brain functional network, ultimately achieving a recognition accuracy rate of 96.50% between normal and depressed individuals. Li et al. (2023) employed a novel approach that integrated fine-grained EEG signals, graph mutual information maximization techniques, and a pre-trained GCN. The innovative method aimed to explore the enhanced interaction among subjects through multi-channel EEG signals, providing a unique perspective for analyzing brain activity patterns. Zhang et al. (2024) employed attention mechanism-based GCNs and LSTM models to detect depression. The integration of existing research with GCNs has typically yielded promising and satisfactory classification outcomes. In this paper, we propose a novel method for depression prediction based on spatial and temporal characteristics. This method utilizes differential entropy (DE) to assess the complexity of EEG signals and characterizes the time-frequency complexity characteristics of brain activity. Simultaneously, a Bidirectional Long Short-Term Memory (BiLSTM) network is introduced to further extract the temporal features of EEG signals. Additionally, the Pearson correlation coefficient is also constructed to evaluate the spatial feature correlation between different EEG channels. Finally, by training and validating the GCN using the extracted time-frequency complexity features and the brain network adjacency matrix based on inter-channel correlations, the ultimate depression classification is achieved. We have abbreviated this strategy that combines DE, BiLSTM, and the GCN network as DBGCN.

The remainder of this article is structured as follows. Section 2 offers a comprehensive overview of the dataset and details the proposed framework. Section 3 presents the experimental setup and results obtained from EEG depression recognition. Lastly, Section 4 summarizes our findings and offers concluding remarks.

## 2 Materials and methods

### 2.1 Subjects

In this study, public datasets were used, including MODMA and PRED+CT. In the MODMA dataset, soft labels are derived from the Patient Health Questionnaire-9 (PHQ-9) score and Generalized Anxiety Disorder-7 (GAD-7), whereas for the PRED+CT dataset, the Beck Depression Inventory (BDI) score is utilized for the derivation of soft labels. Table 1 describes the datasets in detail.

**Table 1:** Properties of the utilized datasets.

Properties	MODMA dataset	PRED+CT dataset
No. of participants	53	119
No. of depression cases (Male/female ratio)	24 (13/11)	44 (12/32)
No. of health cases (Male/female ratio)	29 (20/9)	75 (35/40)
Depression diagnostics	Diagnosis	BDI + Diagnosis
No. of channels	128	64
Sampling rate, Hz	250	500

#### 2.1.1 MODMA dataset

The publicly available dataset provided by Cai et al. (2020) was utilized to evaluate the depression prediction method proposed in this study. The dataset, published by the UAIS laboratory of Lanzhou University in 2020, contains EEG data from patients with clinical depression as well as data from normal controls. The EEG dataset includes resting EEG signals collected from 53 subjects using the HydroCel Geodesic Sensor Net (HCGSN) with 128 channels. The 53 participants consisted of 24 major depressive patients and 29 normal controls. The sampling rate was 250 Hz.

#### 2.1.2 PRED+CT dataset

The other dataset used in this study is available on the PRED+CT website (Cavanagh et al., 2017) and originally contained EEG signals from 121 subjects with an average age of 18.86 ± 1.19 years. However, two subjects’ practical information was missing and was subsequently removed from the dataset (Cavanagh et al., 2018). This study was conducted involving 44 subjects with depression (12 males and 32 females) having high BDI scores (≥13) and 75 control subjects (35 males and 40 females) having low BDI scores (<7). All participants were carefully selected to ensure they had no prior history of head trauma, epileptic seizures, or psychoactive medication usage. The data was collected using a 64-channel EEG system with electrode settings based on the 10–20 standards for EEG recording. The sampling frequency was set at 500 Hz during the resting state. All participants provided written consent approved by the University of Arizona. The subjects had no history of head trauma or seizures. They were not taking any psychoactive medications. Participants were recruited from introductory psychology courses based on their BDI scores.

### 2.2 Data preprocessing

The acquisition of EEG signals is susceptible to disruptions caused by inadvertent human handling, external environmental interferences, and electromagnetic disturbances originating from the device. These factors can introduce various types of noise into the collected data. Although the amplifier within the acquisition equipment can mitigate the impact of certain interference noises, a range of endogenous and exogenous artifacts may persist. Exogenous artifacts are mainly caused by power frequency interference. Endogenous artifacts are mainly caused by interference from electrocardiogram (ECG), electromyography (EMG), and electrooculography (EOG) that overlap with EEG within the frequency band (Walczak and Chokroverty, 1994). Therefore, to obtain relatively pure EEG signals, preprocessing is necessary.

The detailed preprocessing of EEG data in this study involves the sequential execution of the following steps:

#### 2.2.1 Filtering

To mitigate the influence of the power grid effect, the EEG signals undergo a notch filtering process at 50 Hz, as per the established protocols (Ding et al., 2019; Zając and Paszkiel, 2020). Subsequently, a bandpass filter is applied, with cut-off frequencies precisely set at 0.3 Hz and 50 Hz. Furthermore, a Butterworth filter of order 4 is employed, having a high cut-off frequency at 50 Hz and a low cut-off frequency at 1 Hz, following the recommendations of Kamenov et al. (2016). This sequential filtering approach allows for the elimination of residual high-frequency noise and low-frequency artifacts while preserving the integrity of critical frequency bands intrinsic to EEG activity. This ensures the enhanced quality and reliability of the EEG data for subsequent analytical purposes.

#### 2.2.2 Baseline correction

Baseline correction serves as a crucial preprocessing step in EEG analysis, aiming to eliminate the direct current (DC) offset that arises during signal recording. This offset, if uncorrected, can significantly impact the accuracy and comparability of EEG signals, introducing bias and distortion. By applying baseline correction, the DC offset is removed, ensuring that the mean of the EEG signal is centered at zero. This normalization step is essential for the accurate interpretation and comparison of EEG data across studies and experimental conditions. We have chosen a baseline period of 120 s, which corresponds to the first 2 min of the selected data.

#### 2.2.3 Artifact removal

Independent Component Analysis (ICA) is employed on the filtered EEG signals to eliminate residual undesired components. The MNE-python package is utilized in this study, leveraging a semi-automatic ICA method for contaminant resolution. The fast ICA algorithm is employed explicitly due to its efficiency compared to traditional ICA methods and its ability to maximize non-Gaussianity. Subsequently, MNE is utilized for artifact detection. The remaining ICA components are back-projected into the channel space (Ablin et al., 2018).

#### 2.2.4 Data segmentation

In this study, a window length of 4 s was chosen, as it offers an optimal balance between capturing representative brain activity patterns and maintaining temporal resolution. Additionally, the sliding length, which governs the overlap between adjacent segments, was set to 2 s. This results in a 50% overlap between consecutive windows, ensuring continuous coverage of brain activity throughout the segments. This approach ensures both comprehensiveness and precision in the analysis of EEG data.

**Figure 1:**
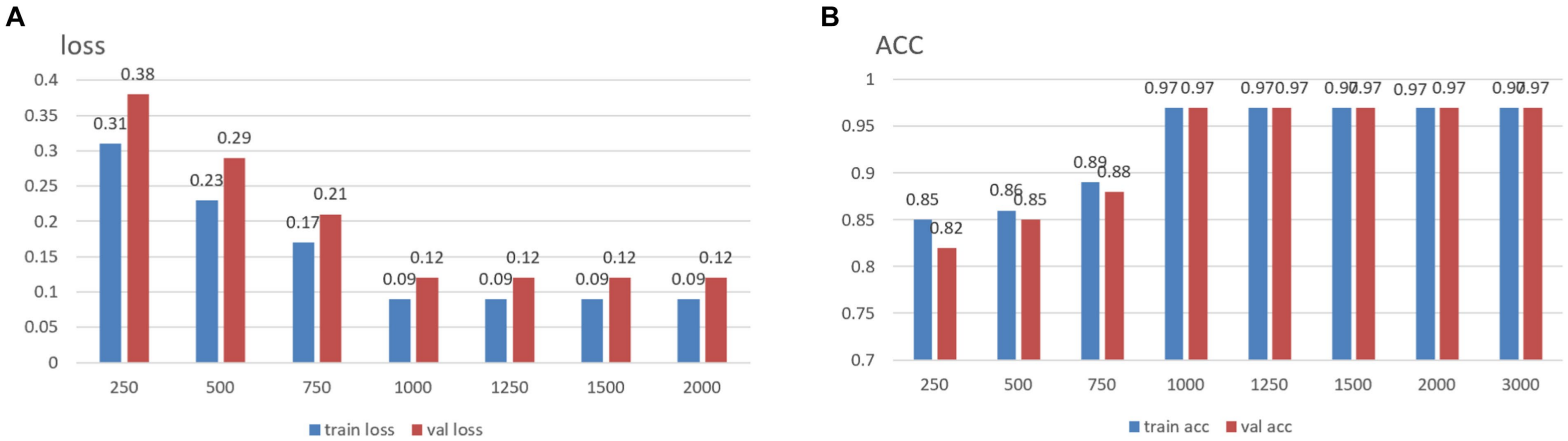
Summary results for datasets of different lengths: **(A)** loss function, **(B)** accuracy.

It is well established that increased data points within a single sample augment its informational content. Conversely, given that the total number of data points in the original dataset is fixed excessively, elongating each sample’s size may result in a reduced number of total samples, thereby impeding the effective training of neural networks. To address this, and to determine an optimal sample length, segments of data from the MODMA dataset were selected and intercepted at lengths of 250, 500, 750, 1,000, 1,250, 1,500, 2000, and 3,000, respectively, forming a smaller dataset. The outcomes for different sample lengths are presented in Figure 1.

The analysis results indicate that increased individual sample points in the data correspond to higher training accuracy, thus confirming the previously proposed speculation. It is noteworthy that, upon reaching a threshold of 1,000 data points per sample, subsequent increases in the dataset size do not yield further improvements in accuracy. In light of dataset size considerations, the desired accuracy, and the smoothness of the loss function curve obtained during training, the decision was made to opt for a sample size of 1,000 data points. We use 50% overlap to ensure continuity and stability in signal processing, avoiding signal disruption and distortion and improving data consistency and reliability.

Taking into account that participants might initially have some time to reflect, yet may experience fatigue, irritability, and other states towards the latter stages of the experiment, we have elected to use intermittent data from these continuous recordings as our experimental dataset. Specifically, for both the PRED+CT and MODMA datasets, we have selected 122 s of persistent EEG recordings for detailed analysis.

In this study, each EEG sample was segmented into 4-s intervals with 50% overlap. This approach yielded a total of 7,140 samples for the PRED+CT dataset and 3,180 samples for the MODMA dataset, respectively. This method ensures that our analysis captures both transient and sustained neural activity patterns across the entire duration of the experiments.

### 2.3 Proposed classification method

**Figure 2:**
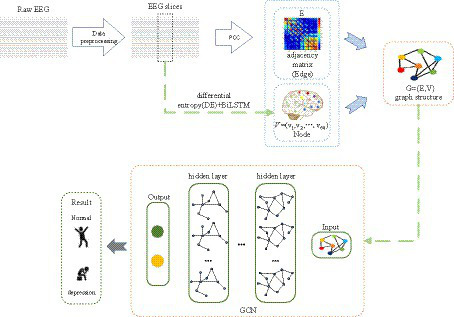
The proposed approach for depression prediction.

Many prior studies have demonstrated that deep learning and EEG can be employed for depression identification (Li et al., 2019; Almars, 2022). As we are aware, EEG signals encompass spatial topological information, yet this facet is often underestimated. Figure 2 provides an overview of the proposed framework for depression prediction. As depicted in Figure 2, the proposed method consists of three steps: feature extraction, adjacency matrix construction, and Graph Convolutional Network (GCN) classification. In the first step, time-frequency complexity features, temporal features, and frequency features of EEG signals are extracted, generating node features for the Graph Convolutional Network. Subsequently, the brain network adjacency matrix is computed based on inter-channel correlations. Finally, by training and validating the Graph Convolutional Network with graph node features and the brain network adjacency matrix based on inter-channel correlations, the ultimate depression classification is achieved.

In the validation experiment, we used 10-fold cross-validation to evaluate the performance of the classifier. During the 10-fold cross-validation process, we divided all subjects’ EEG data into 10 equal portions. In each iteration, nine portions were selected as the training set, while the remaining portion served as the validation set, ensuring that every subset was validated. It is important to note that we did not mix the EEG segments from all subjects; instead, we maintained the integrity of each subject’s data. This approach better simulates real-world scenarios and assesses the model’s generalization ability across different subjects.

#### 2.3.1 Feature extraction

DE extends the principles of Shannon entropy and is employed for evaluating the complexity of a continuous random variable (Duan et al., 2013; Shi et al., 2013; Chen et al., 2019). Given the characteristic of EEG data, which manifests higher energy in lower frequencies in contrast to higher frequencies, DE provides a capability to discern between patterns of low and high-frequency energy in EEG data. The initial application of DE in emotion recognition based on EEG was introduced by Duan et al. (2013). Empirical evidence has demonstrated that, within a fixed-length EEG data segment, Differential Entropy (DE) is equivalent to the logarithm energy spectrum in a specific frequency band (Shi et al., 2013). Consequently, following segment enhancement, the time-series data undergoes decomposition using a fourth-order Butterworth filter into four distinct frequency bands, to capture neurophysiological patterns associated with specific cognitive or physiological processes. The EEG signals are decomposed into four distinct frequency bands: delta (0.5–4 Hz), theta (4–8 Hz), alpha (8–12 Hz), and beta (12–30 Hz). This selection is based on standard EEG analysis, thereby facilitating the identification of frequency signatures pertinent to the study. The DE features of the four frequency bands are then calculated to characterize the time-frequency domain complexity of the EEG signals. The DE calculation formula for each channel is shown in Eq. 1.

(1) $$X=\frac{1}{2}\ln\left(P_{i}\right)+\frac{1}{2}\ln\left(\frac{2\pi e}{N}\right)$$

Where *P_i_* is the entropy of a certain frequency band of EEG, *N* is the time length. *X* represents the features derived from DE.

Then, all DE features are normalized before input into the BiLSTM network to capture the dynamic characteristics of the EEG signals over time and further obtain the time-series features of the EEG signals. The resulting features are employed as node input for the graph convolutional network. The calculation formula for graph node features is given by Eq. 2.

(2) $$H=\mathrm{BiLSTM}\;\left(X\right)$$

Where $X\in R^{n\times t\times d}$ represents the features derived from DE, and $H\in R^{n\times d^{\prime }}$ denote the resulting graph node features that encompass both temporal and frequency-domain information. Here, n represents the number of channels, t denotes the time sequence, and d is the number of spectral components. 𝑑′ signifies the output dimension of the BiLSTM.

#### 2.3.2 Spatial topological structure

Utilizing the Pearson correlation coefficients extracted between channels as spatial topological features of EEG signals, it measures the spatial correlation or connectivity strength between different brain regions.

Given two random EEG channels X and Y, the formula for calculating the Pearson correlation coefficient is shown in Eq. 3 (Hasan et al., 2020):

(3) $$r=\frac{\sum_{i=1}^{n}\left(X_{i}-\bar{X}\right)\left(Y_{i}-\bar{Y}\right)}{\sqrt{\sum_{i=1}^{n}{\left(X_{i}-\bar{X}\right)}^{2}}\sqrt{\sum_{i=1}^{n}{\left(Y_{i}-\bar{Y}\right)}^{2}}}$$

Where n is the number of samples, X_i_ and Y_i_ are the single sample values of X and Y, $\bar{X}$ is the sample mean value of X, and $\bar{Y}$ is the sample mean value of Y. If r is more significant than zero, it indicates a positive correlation between two vectors; if the value is less than zero, the vectors are negatively correlated; if the value is equal to zero, the vectors are uncorrelated. If r equals one, X and Y can be described by a linear equation, where all data points fall on a straight line, and Y increases with the increase in X, indicating a linear positive correlation. On the other hand, if r takes a value of negative one, all data points still lie on the same straight line, but this time, Y decreases with the increase in X, indicating a linear negative correlation.

**Figure 3:**
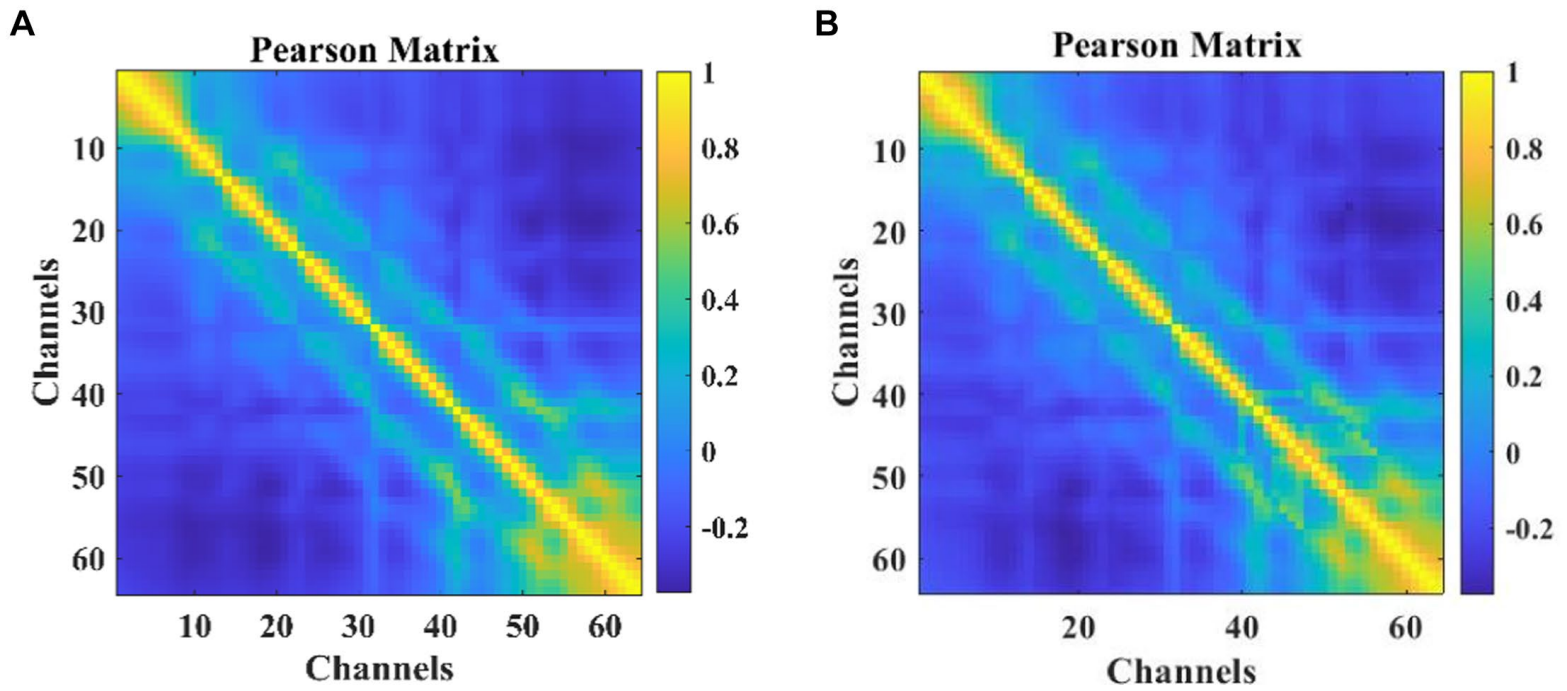
The heat map of Pearson correlation coefficients among participants: **(A)** MODMA **(B)** PRED+CT.

Correlation calculations were performed on the EEG data from 64 channels, resulting in a matrix A of size 64 × 64 as shown in Eq. 4. The heatmap visualization is illustrated in Figure 3, where strong correlations between channels can be observed. To ensure data consistency, experiments were conducted using only the corresponding data from 64 channels, even though 64 channels were available.

(4) $$A=\left[\begin{array}{ccc} a_{\left(1,1\right)} & \cdots & a_{\left(1,64\right)}\\ \vdots & \ddots & \vdots \\ a_{\left(64,1\right)} & \cdots & a_{\left(64,64\right)} \end{array}\right]$$

Each element $a_{\left(i,j\right)}\left(i=1,2,\;\cdots \;,64;\;j=1,2,\;\cdots \;,64\right)$ in matrix A represents the calculated channel correlation.

#### 2.3.3 GCN network structure

EEG signals are collected through channels distributed across different spatial positions, and the state of each channel and the relationships between channels are crucial for identifying depression. This interplay can be conceptualized as an irregular graph structure, also referred to as a topology. Two essential components are present in graph data: nodal features (data of nodes) and graph structure (connections between nodes). It’s worth noting that the structure around each node may be unique. The flexibility and complexity of this data structure render traditional Convolutional Neural Networks (CNNs) less advantageous. Consequently, we consider shifting convolution operations from dealing with conventional Euclidean structured data to handling graph data with a topological structure. The GCN is a type of convolutional neural network directly applied to graphs, utilizing structural information for feature extraction. Similar to traditional CNNs, it typically comprises convolutional layers, pooling layers, activation functions, fully connected layers, and other integral components.

There is a graph with N nodes. The input feature dimension of each node is D, and the features of all nodes will form an N × D feature matrix H. At the same time, an N × N Adjacency Matrix (A) is formed by analyzing the functional connection relationships among nodes. The inputs to the GCN model are the feature matrix H and the adjacency matrix A. The mode of propagation between layers of GCN is shown in Eq. 5.

(5) $$H^{\left(l+1\right)}=\sigma \left({\tilde{D}}^{-\frac{1}{2}}\tilde{A}{\tilde{D}}^{-\frac{1}{2}}H^{\left(l\right)}W^{\left(l\right)}\right)$$

σ is a nonlinear activation function. $\tilde{D}$ is the degree matrix of $\tilde{A}$ . $\tilde{A}$ *=*A + I, where I is the identity matrix. A is an adjacency matrix of one of the inputs to the model, and W is the weight matrix to be trained.

**Figure 4:**
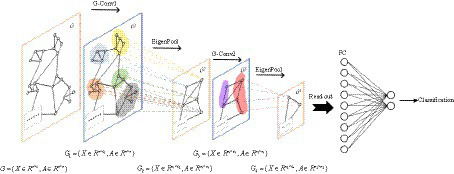
GCN classification algorithm process.

We choose BReLU as the non-linear activation function. Using spectral pooling operation to reduce the size of the graph, based on experience, this article selects 50% spectral pooling, which means reducing the number of nodes in each pooling by half. The fully connected layer uses the softmax function to perform the final binary classification. The GCN model is illustrated in Figure 4.

The loss function uses a cross-entropy loss function with L2 regularization is shown in Eq. 6:

(6) $$\mathrm{loss}=-\overset{2}{\underset{i=1}{\sum }}y_{i}\log\left(y_{i}\right)+\lambda ∥\overset{n}{\underset{j=1}{\sum }}w_{j}^{2}+b_{j}^{2}∥$$

Where λ is the regularized penalty coefficient for L2, $w_{j}$ , $b_{j}$ is the model parameter, and n is the number of input samples.

To find the most optimal parameter that makes the value of the loss function as small as possible, this paper conducts a comparative experiment on the MODMA dataset for depression recognition. The accuracy and loss rates of the four optimizers were compared for different Epochs, and the results are shown in Tables 2, 3, where it can be seen that the Adam optimizer performed the best.

**Table 2:** Classification accuracy of different optimizers under different epoch (Accuracy).

Optimizer	Epoch = 50		Epoch = 100		Epoch = 150	
	Training set accuracy	Test set accuracy	Training set accuracy	Test set accuracy	Training set accuracy	Test set accuracy
SGD	81.46%	80.02%	87.37%	86.25%	86.87%	86.11%
Momentum	86.24%	84.78%	91.21%	89.29%	92.21%	91.29%
RMSProp	89.5%	89.99%	94.67%	94.14%	92.67%	92.14%
Adam	91.3%	90.88%	97.88%	98.30%	96.68%	95.30%

**Table 3:** Cross entropy loss of different optimizers under different epoch (Loss).

Optimizer	Epoch = 50		Epoch = 100		Epoch = 150	
	Training set loss	Test set loss	Training set loss	Test set loss	Training set loss	Test set loss
SGD	30.82%	37.56%	28.30%	32.46%	28.31%	32.35%
Momentum	27.34%	27.63%	15.21%	22.71%	15.11%	22.65%
RMSProp	14.90%	17.54%	13.75%	14.13%	13.65%	14.07%
Adam	11.22%	11.35%	9.52%	12.20%	9.52%	12.20%

#### 2.3.4 Network parameters

In this study, the scikit-learn based grid search method (Cong et al., 2021) was chosen to tune the network parameters and hyperparameters to determine the best combination of parameters for the model. The grid search method has higher efficiency and faster efficiency than the random search and Bayesian optimization methods.

The procedure of selecting the optimal combination of hyperparameters to improve a model’s performance is referred to as hyperparameter tuning. In neural network models, Epoch and Batchsize are two particularly significant hyperparameters. Our study employs the grid search method to fine-tune these parameters, utilizing the optimal combination obtained from adjusting network parameters. As depicted in Table 4, the model attains its peak performance with an accuracy of 0.9830 (highlighted in bold in the table) when the Epoch is set to 100 and the Batchsize is set to 512, shown as bold values. Other related core hyperparameters are the learning rate, which is 0.00001, and the dropout, which is 0.5.

**Table 4:** Hyperparameter tuning.

Model	Epoch	Batchsize	Accuracy
M1	100	256	0.8871
M2	200	256	0.9023
M3	300	256	0.9101
**M4**	**100**	**512**	**0.9830**
M5	200	512	0.9687
M6	300	512	0.9564
M7	100	1,024	0.9135
M8	200	1,024	0.9044
M9	300	1,024	Saturated

## 3 Results and discussion

We propose a new network model that combines DE, BiLSTM, and GCN in this article, and it has contributions in two aspects: 1) Considering the time-frequency complexity of EEG signals, DE is calculated after dividing the EEG signals into different frequencies, which is used as a feature of the data. A BiLSTM network is introduced to extract the temporal features of EEG signals further; 2) The Pearson correlation coefficient is calculated to evaluate the spatial feature correlation between different EEG channels and construct a topological map.

To affirm the reliability and generalizability of the classifiers and datasets, we select the accuracy and Confusion matrix to evaluate the performance of the model. The experimental environment was an Inter(R) Core i7-10875H CPU and NVIDIA RTX 2060 GPU. All experiments are implemented using MATLAB R2021b and Python 3.7. Evaluation Metrics.

(1) Accuracy is defined as the ratio of correctly classified samples to the total number of samples within a given test dataset. Its formula is shown in Eq. 7.

(7) $$\mathrm{Accuracy}=\frac{\left|TP\right|+\left|TN\right|}{\left|TP\right|+\left|FP\right|+\left|TN\right|+\left|FN\right|}$$

In the context of depression recognition, the terminology is as follows: True Positive (TP) denotes the count of samples accurately predicted as depressed and indeed exhibiting depression; False Positive (FP) signifies the count of samples erroneously indicated as depressed but, in reality, being healthy; True Negative (TN) corresponds to the count of samples correctly predicted as healthy and indeed exhibiting a healthy state; False Negative (FN) pertains to the count of samples inaccurately predicted as healthy, yet manifesting depression.

(2) Confusion matrix is also an effective model evaluation index, which can more intuitively show the classification accuracy of the dataset. The horizontal axis represents the predicted values, while the vertical axis represents the true values.

**Figure 5:**
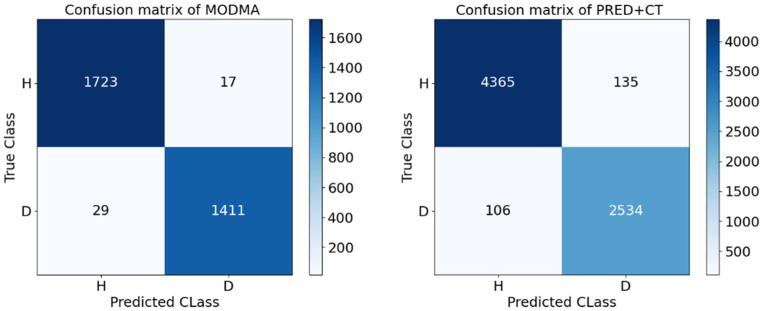
Confusion matrix on DE feature of MODMA and PRED+CT.

Table 5 presents the accuracy and standard deviation of five different methods on the MODMA dataset across four frequency bands and all frequency bands, respectively. We compare the classification accuracy and standard deviation of SVM (Wang et al., 2011), GCN, LSTM, BiLSTM, GTSAN (Yang et al., 2023), and our proposed DBGCN method. According to the table, it is evident that our proposed DBGCN method outperforms the other methods in terms of classification accuracy and standard deviation. It can be seen that:

1. The recognition accuracy of all methods in the δ, θ, α, and full frequency bands are significantly higher than that in the β band. This suggests a strong correlation between the low-frequency band of EEG and depression.
2. In most deep neural networks, the accuracy of the entire frequency band is higher than that of a subband, indicating that the entire frequency band provides a more comprehensive and effective representation compared to a single subband.
3. Compared to other methods, the method proposed in this paper exhibits significantly higher accuracy in the feature DE. The accuracy achieved in the δ, θ, α, β, and full frequency bands are 94.10, 93.38, 94.27, 80.28, and 98.30%, respectively. The method proposed in this paper is significantly more accurate than other methods in feature DE. The accuracy on DE achieves 94.10, 93.38, 94.27, 80.28, and 98.30 in the δ, θ, α, β, and full frequency bands, respectively.
4. The standard deviations of DBGCN are minor compared to those of other methods, indicating that DBGCN has better stability.
5. To demonstrate the effectiveness of depression classification using DE features, we present confusion matrices for the MODMA and PRED+CT datasets in Figure 5. The vertical axis represents the true label, while the horizontal axis represents the label predicted by the model. As shown in Figure 5, the proposed method achieves high classification accuracy on both datasets.

**Table 5:** Comparison of average accuracy and standard deviation of accuracy on the MODMA dataset.

Feature	Classifier	δ (%)	θ (%)	α (%)	β (%)	Full bands (%)
DE	SVM (Wang et al., 2011)	80.76 ± 11.38	79.55 ± 10.51	77.54 ± 11.68	60.85 ± 14.37	84.99 ± 9.71
	GCN	82.75 ± 10.23	83.45 ± 11.34	81.23 ± 8.56	63.43 ± 12.35	86.56 ± 9.21
	LSTM	85.43 ± 9.65	85.23 ± 10.03	84.43 ± 11.26	73.20 ± 16.61	90.23 ± 8.37
	BiLSTM	90.38 ± 8.67	90.61 ± 8.65	90.11 ± 8.14	78.11 ± 15.37	92.11 ± 8.35
	GTSAN (Yang et al., 2023)	92.38 ± 7.34	91.27 ± 7.03	90.24 ± 7.06	82.13 ± 14.31	96.85 ± 4.63
	BiLSTM + GCN	94.10 ± 6.75	93.38 ± 7.76	94.27 ± 6.11	80.28 ± 11.75	98.30 ± 3.63
PSD	SVM (Wang et al., 2011)	70.96 ± 11.21	79.38 ± 11.37	78.48 ± 10.93	63.75 ± 12.53	82.39 ± 9.27
	GCN	81.79 ± 9.38	80.45 ± 10.31	78.39 ± 9.75	69.43 ± 11.28	85.21 ± 8.36
	LSTM	83.51 ± 10.34	82.59 ± 7.08	80.83 ± 10.17	70.81 ± 11.62	89.91 ± 9.83
	BiLSTM	90.01 ± 10.35	87.0.91 ± 10.43	88.60 ± 10.03	64.53 ± 12.37	91.15 ± 9.18
	GTSAN (Yang et al., 2023)	90.37 ± 7.67	91.66 ± 7.98	91.89 ± 7.90	87.89 ± 9.46	97.56 ± 3.37
	BiLSTM + GCN	92.36 ± 5.65	91.38 ± 7.63	91.65 ± 6.55	82.57 ± 7.67	96.30 ± 3.87

When tested on the depression recognition task using the MODMA dataset with DE features, our results are not only superior to traditional deep learning methods but also exhibit a higher classification accuracy than popular deep learning algorithms in recent years. As shown in Table 5, the classification accuracy of DBGCN is nearly 1.5% higher than that of the GTSAN method.

**Figure 6:**
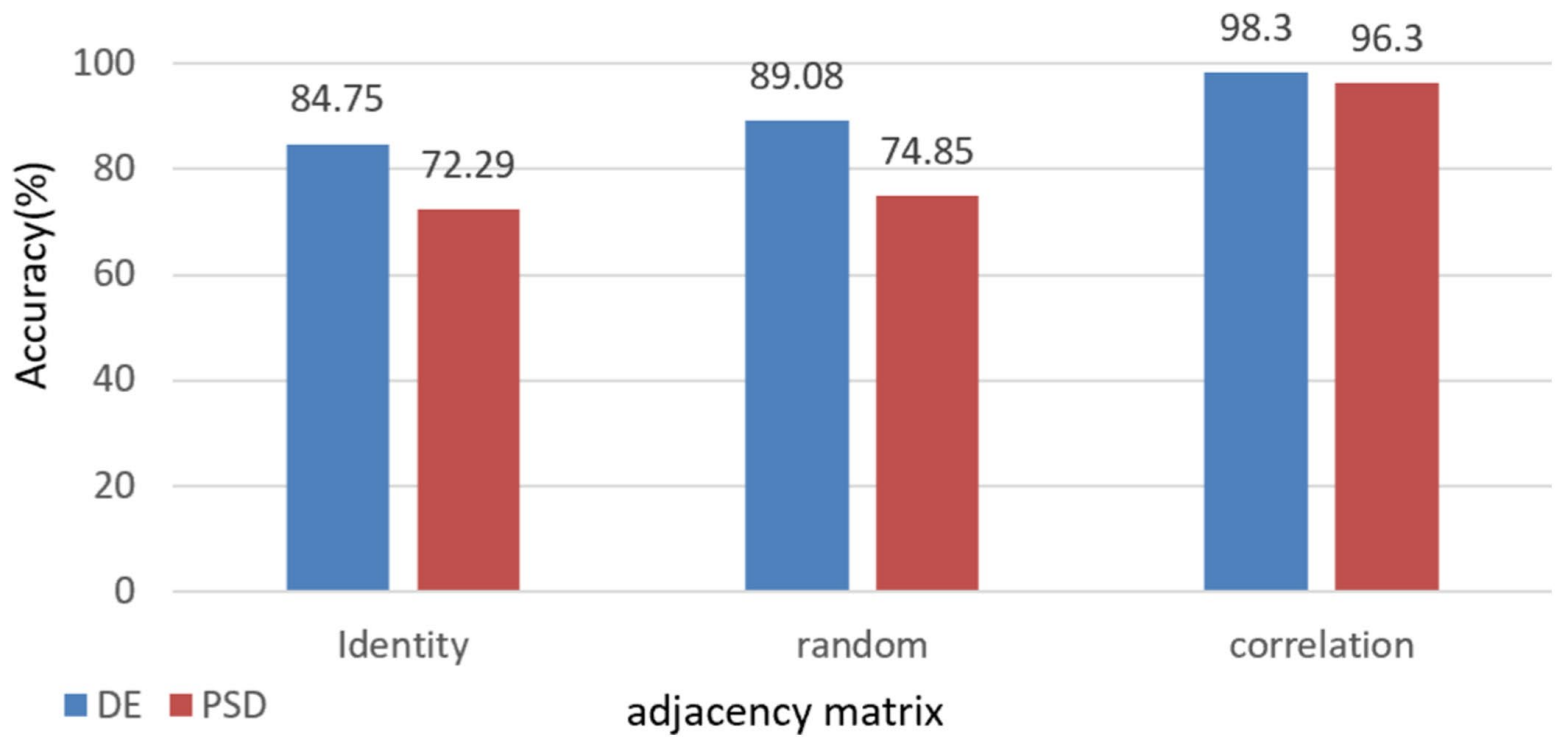
Impact of different adjacency matrices on model performance with comparative analysis of full-band DE & PSD Features.

To further demonstrate the validity and necessity of this model, we conducted an ablation experiment. We replaced the adjacency matrix in the network with an identity matrix and a random matrix for comparison. These substitutions were applied to the full frequency bands of DE and PSD, respectively, with the results presented in Figure 6. These results indicate that using the correlation matrix achieved higher scores than the identity and random matrices. This is because only the correlation matrix takes into account the structural relationship among EEG channels.

We computed the average estimate of measurement accuracy for each model on the PRED+CT dataset. As shown in Table 6, the DBGCN model demonstrates optimal performance. Shallow models, such as SVM, exhibit accuracies below 85%. In contrast, DBGCN achieves an accuracy surpassing 98%. This observation highlights the capability of deep learning methods to extract discriminative features automatically.

**Table 6:** Comparison of average accuracy and standard deviation of accuracy on the PRED+CT dataset.

Feature	Classifier	Full bands(%)
**DE**	SVM (Wang et al., 2011)	82.79 ± 8.71
	GCN	88.75 ± 7.18
	LSTM	89.19 ± 7.54
	BiLSTM	90.37 ± 7.26
	GTSAN (Yang et al., 2023)	94.47 ± 3.54
	BiLSTM + GCN	96.51 ± 4.12
PSD	SVM (Wang et al., 2011)	80.23 ± 7.14
	GCN	83.92 ± 7.87
	LSTM	88.61 ± 8.57
	BiLSTM	90.55 ± 8.78
	GTSAN (Yang et al., 2023)	95.43 ± 5.07
	BiLSTM + GCN	95.27 ± 4.87

In the context of the MODMA dataset, our investigation involved a thorough comparison of prominent methodologies previously examined on the identical dataset, as delineated in Table 7. Sun et al. (2020) conducted an exhaustive examination of EEG signals emanating from individuals diagnosed with major depression. Their analytical approach encompassed the extraction of diverse feature sets, encompassing the Phase Lag Index (PLI) features, Linear features (L), and Nonlinear features (NL), alongside composite combinations of these features. This systematic exploration aimed to provide a comprehensive understanding of the intricate relationships within the dataset, fostering a nuanced perspective on the discriminative capabilities of the evaluated techniques. The utilization of varied feature types and their amalgamations by Sun et al. contributes to the richness of the feature space under consideration, enhancing the interpretability of subsequent findings. Subsequently, a comprehensive evaluation was conducted employing four distinct classifiers to discern the most efficacious features. Following rigorous experimentation, it was discerned that the PLI derived from functional connectivity features exhibited superior performance compared to alternative feature sets. Notably, the outcomes revealed that the classification accuracy achieved by LR and ReliefF reached a commendable 82.31%. Shen et al. (2021) introduced an optimal channel selection technique denoted as mKTAChSel, founded on kernel-target alignment, specifically designed for the detection of depression through electroencephalogram (EEG) data. Employing this method with a Support Vector Machine (SVM) classifier, they achieved a noteworthy classification accuracy of 81.6% on the MODMA dataset. This underscores the efficacy of their proposed mKTAChSel method in discerning relevant information from EEG signals for precise depression detection, showcasing its potential as a valuable tool in the realm of EEG-based diagnostic approaches. In a recent study, Yang et al. (2023) introduced a model named GTSAN, which utilizes causal convolution and dilated convolution to extract features across a range of scales from fine to coarse. Their model demonstrated significant efficacy, achieving a classification accuracy of 97.56% on the MODMA dataset. In comparison, the strategy proposed in this study yielded even more noteworthy results, with an accuracy of 98.3%. This suggests that the proposed method exhibits a more competitive performance on the MODMA dataset in the context of depression detection.

**Table 7:** The experimental accuracies of different models on the MODMA dataset.

Reference	Methods	Accuracy (%)
Sun et al. (2020)	L + DT	74.20
	NL + kNN	65.42
	L&NL + NB	75.52
	PLI + LR	82.03
	L&PLI + LR	80.99
	NL&PLI + LR	81.79
	ALL + LR	82.31
Shen et al. (2021)	mKTAChSel + SVM	81.60
Yang et al. (2023)	PSD + GTSAN	97.56
Proposed	DE + BiLSTM + GCN	98.30

Furthermore, we have also applied other state-of-the-art deep learning models, such as EEGNet (Lawhern et al., 2018) and GTSAN (Yang et al., 2023), to these two datasets and compared their results with those of the DBGCN model. The comparison results are summarized in Table 8. It can be seen that the DBGCN model, which integrates time-frequency features and spatial topology, achieves 98.30 and 96.51% accuracy on the MODMA and PRED+CT datasets, respectively. These results demonstrate the performance of the DBGCN model in detecting depression.

**Table 8:** The comparison of the accuracies in different references.

References	Dataset	HC/MDD	Methods	Accuracy (%)
Lawhern et al. (2018)	MODMA	29/24	EEGNet	92.33
Lawhern et al. (2018)	PRED+CT	48/71	EEGNet	90.05
Yang et al. (2023)	MODMA	29/24	PSD + GTSAN	97.56
Yang et al. (2023)	PRED+CT	48/71	PSD + GTSAN	96.03
Proposed	MODMA	29/24	DE + BiLSTM + GCN	98.30
Proposed	PRED+CT	48/71	DE + BiLSTM + GCN	96.51

DE features of the EEG signal are extracted, considering the time-frequency complexity of EEG signals. A BiLSTM network is introduced to extract the temporal features of EEG signals further. Calculating the Pearson correlation coefficient to evaluate the spatial feature correlation between different EEG channels and constructing a topological map. Propose a new network model that combines DE, BiLSTM, and GCN.

We validated the model on two datasets. From the obtained experimental results, it is evident that the DBGCN model effectively integrates time-frequency characteristics and spatial topology structures, yielding a remarkable accuracy of 98.30% on the MODMA dataset and 96.51% on the PRED+CT dataset, respectively. This underscores the robust performance of the DBGCN model in detecting depression. Compared to existing methodologies applied to the same datasets, the proposed model demonstrates superior predictive capabilities for depression. The achieved accuracy surpasses that reported in prior studies, affirming the efficacy of the proposed strategy, as briefly summarized in Table 8.

In contrast to prevailing methodologies characterized by manual EEG feature extraction, our approach directly utilizes preprocessed signals without requiring manual feature extraction as the input to the DBGCN model. Remarkably, our method achieves notable accuracies of 98.3 and 96.51% on the MODMA and PRED+CT datasets, respectively. These results suggest the potential of our method to establish an end-to-end depression detection system, indicating its capacity to effectively process raw signals and autonomously discern meaningful patterns for accurate diagnostic purposes.

We used 10-fold cross-validation to validate the model’s efficacy and robustness. In the 10-fold cross-validation procedure, the entire dataset is meticulously partitioned into 10 distinct folds, ensuring an equal distribution of samples across each fold. For each iteration, nine folds serve as the training dataset, within which an additional split of 90 and 10% is made to facilitate model training and hyperparameter tuning, respectively. The remaining one fold is designated as the validation dataset, exclusively used to assess the model’s performance. This entire process is repeated 10 times, ensuring that each fold serves as the validation dataset once, providing a comprehensive and unbiased evaluation of the model’s generalization capabilities.

The performance metrics for each fold of the 10-fold cross-validation are presented in Table 9. As per the methodology outlined by Sokolova and Lapalme (2009), precision signifies the model’s capacity to avoid misclassifying negative samples (MDD patients) as positive samples (healthy controls), sensitivity denotes the model’s accuracy in correctly identifying positive healthy samples, and specificity represents the model’s accuracy in correctly identifying negative MDD samples. Our study has achieved notably high-performance metrics exceeding 96.5%, with a minimal standard deviation of less than 1.

**Table 9:** Summary of various performance parameters (%) obtained with 10-fold cross-validation strategy.

Fold	Accuracy(%)	Precision(%)	Sensitivity(%)	F1-score(%)
1	97.08	96.87	96.95	96.94
2	96.46	95.78	95.95	96.42
3	96.74	96.9	95.99	96.6
4	96.07	96.03	96.11	96.6
5	96.74	96.76	96.07	96.22
6	95.65	97.08	97.21	96.49
7	96.48	96.90	96.67	96.45
8	96.78	96.30	96.14	96.7
9	96.76	96.65	97.18	96.85
10	96.44	95.53	96.83	95.73
Average	96.52	96.48	96.51	96.50
Standard deviation	0.39	0.51	0.48	0.33

## 4 Conclusion

This paper designs a novel model approach for predicting depression emotions. The proposed method extensively extracts both time-frequency information and time-frequency complexity features from the raw signals. Considering the correlation between electrodes in the data acquisition equipment, spatial topological features are extracted using graph convolutional networks for final classification. To assess the model’s effectiveness and stability, we employed a 10-fold cross-validation approach. The proposed strategy outperforms other models applied to the MODMA and PRED+CT datasets.

By utilizing DE, BiLSTM, and graph convolution, we propose a depression emotion recognition model based on DBGCN for EEG signals. Constructing an adjacency matrix using Pearson correlation coefficients allows us to capture the inter-channel correlations in EEG signals. DE and BiLSTM are then utilized to extract the time-frequency features of EEG signals. Consequently, the features extracted by the neural network contribute to a more effective improvement in the accuracy of depression emotion classification. Test results on the public datasets MODMA and PRED+CT demonstrate that, compared to other models, DBGCN can more accurately classify depression emotions based on the feature DE. Particularly noteworthy is its superior performance when handling the entire frequency band. Additionally, we leverage the Pearson correlation coefficient matrix to demonstrate the significant impact of inter-channel correlations in EEG signals on the network’s predictive outcomes.

The outcomes emphasize the effectiveness of the proposed model in the context of EEG-based depression classification tasks, thereby indicating its potential for integration into future diagnostic processes for depression recognition. However, transitioning this model into clinical practice requires a significant refinement process. A model must exhibit exceptional accuracy and robustness across diverse patient populations to be clinically feasible. Although our model has been validated on two public datasets, further validation with additional large and representative datasets is crucial to ensure its safety and efficacy in clinical settings. Furthermore, given its promising performance, the proposed model could also be explored for its application in the auxiliary diagnosis of other psychiatric disorders.

Additionally, the analysis in this study predominantly relies on EEG data, and the clinical interpretability is somewhat limited. Moving forward, we intend to collaborate with hospitals to enhance clinical interpretability by incorporating expert knowledge.

## Data availability statement

The original contributions presented in the study are included in the article/supplementary material, further inquiries can be directed to the corresponding author.

## Author contributions

WL: Data curation, Investigation, Methodology, Software, Validation, Visualization, Writing – original draft, Writing – review & editing. KJ: Conceptualization, Supervision, Writing – review & editing. ZW: Project administration, Writing – review & editing.
